# Functional characterization of maize heat shock transcription factor gene *ZmHsf01* in thermotolerance

**DOI:** 10.7717/peerj.8926

**Published:** 2020-04-10

**Authors:** Huaning Zhang, Guoliang Li, Dong Hu, Yuanyuan Zhang, Yujie Zhang, Hongbo Shao, Lina Zhao, Ruiping Yang, Xiulin Guo

**Affiliations:** 1 Plant Genetic Engineering Center of Hebei Province/Institute of Genetics and Physiology, Hebei Academy of Agriculture and Forestry Sciences, Shijiazhuang, China; 2 College of Life Science, Hebei Normal University, Shijiazhuang, Hebei, China; 3 College of Agriculture and Forestry Science and Technology, Hebei North University, Zhangjiakou, China; 4 Salt-soil Agricultural Center, Key Laboratory of Agricultural Environment in the Lower Reaches of Yangtze River Plain, Institute of Agriculture Resources and Environment, Jiangsu Academy of Agricultural Sciences (JAAS), Nanjing, P.R. China; 5 Jiangsu Key Laboratory for Bioresources of Saline Soils, Jiangsu Synthetic Innovation Center for Coastal Bio-agriculture, Yancheng Teachers University, Yancheng, Jiangsu, China; 6 College of Environment and Safety Engineering, Qingdao University of Science & Technology, Qingdao, China

**Keywords:** Thermotolerance, Heat shock transcription factors, Maize, ABA, Subcellular localization, H_2_O_2_, Adversity stresses, Signal transduction pathway, Functional analysis, *ZmHsf01*

## Abstract

**Background:**

Heat waves can critically influence maize crop yields. Plant heat shock transcription factors (HSFs) play a key regulating role in the heat shock (HS) signal transduction pathway.

**Method:**

In this study, a homologous cloning method was used to clone HSF gene *ZmHsf01* (accession number: MK888854) from young maize leaves. The transcript levels of *ZmHsf01* were detected using qRT-PCR in different tissues and treated by HS, abscisic acid (ABA), hydrogen peroxide (H_2_O_2_), respectively, and the functions of gene* ZmHsf01* were studied in transgenic yeast and *Arabidopsis*.

**Result:**

*ZmHsf01* had a coding sequence (CDS) of 1176 bp and encoded a protein consisting of 391 amino acids. The homologous analysis results showed that ZmHsf01 and SbHsfA2d had the highest protein sequence identities. Subcellular localization experiments confirmed that ZmHsf01 was localized in the nucleus. *ZmHsf01* was expressed in many maize tissues. It was up-regulated by HS, and up-regulated in roots and down-regulated in leaves under ABA and H_2_O_2_treatments. *ZmHsf01*-overexpressing yeast cells showed increased thermotolerance. In *Arabidopsis* seedlings, *ZmHsf01* compensated for the thermotolerance defects of mutant *athsfa2*, and* ZmHsf01*-overexpressing lines showed enhanced basal and acquired thermotolerance. When compared to wild type (WT) seedlings, *ZmHsf01*-overexpressing lines showed higher chlorophyll content and survival rates after HS. Heat shock protein (HSP) gene expression levels were more up-regulated in *ZmHsf01*-overexpressing *Arabidopsis* seedlings than WT seedlings. These results suggest that *ZmHsf01* plays a vital role in response to HS in plant.

## Introduction

High temperature is an abiotic stress factor with a great impact on crop yields and quality. The North China Plain’s typical temperature and monsoonal climate means that agricultural production is often subjected to the frequency and intensity of heat waves (Li et al., 2018; Suchul & Eltahir, 2018). High temperature and drought stress often cause declines in the production and quality of maize, particularly during the early stages of the growth period (Cairns et al., 2012). Controllable and appropriate heat acclimation can induce related gene expression and the synthesis of heat shock proteins (HSPs) and protective enzymes (Nover et al., 1996), so as to help plants obtain thermotolerance and adjust to the even more extreme high temperatures. Studies have shown that heat shock transcription factors (HSFs) have a regulatory role in the thermotolerance-formation process. HSFs can bind to heat shock elements (HSEs) on the promoter regions of HSPs or other related genes and can activate downstream genes to generate a heat shock (HS) response (Nover et al., 2001). Therefore, HSFs have been proved to be important regulators in transcriptional activation, especially in the signal transduction pathway activated by heat and other stresses (Aranda et al., 1999).

Several HSFs have been cloned from different species since the yeast HSF was first isolated in the 1980s (Baniwal et al., 2004; Bharti et al., 2004; Czarnecka-Verner et al., 2004; Kotak et al., 2004; Zhu et al., 2006; Guo et al., 2008; Yang et al., 2016). The first plant HSF was cloned from tomato in 1990 (Yokotani et al., 2008). The HSF multi-gene family is divided into three classes (A, B, and C) based on gene structures, and each class contains different subclasses. The gene number of the HSF family also varies. There is only one HSF gene in the fruit fly and yeast, and vertebrates have at least four HSFs (Nakai, 2016). Plants possess more HSFs than other organism. To date, 21 HSFs in *Arabidopsis* and 24 HSFs in tomato have been identified (Scharf et al., 2012), and at least 56 HSFs in wheat have been predicted (Xue et al., 2014).

Few functional analyses were reported on plant HSFs, and previous studies were limited to analysis of the A1 and A2 subclasses of *Arabidopsis* and tomato. Previous studies showed that the tomato *HsfA1a* is constitutively expressed and that the deduced protein is localized in both nucleus and cytoplasm under normal growth conditions (Scharf et al., 1998). In tomato, *HsfA2* and *HsfB1* synthesis, which induces HSPs expression, is regulated by HsfA1a for HS resistance (Scharf et al., 1998; Liu et al., 2013). Strong and stable *HsfA2* is up-regulated by heat induction, and a considerable *HsfA2* accumulation appears during late and recovery periods after heat stress in tomato (Heerklotz, Döring & Bonzelius, 2001). Because of strong cytoplasmic localization signals, the nuclear localization of *HsfA2* has to rely on the heterooligomer formed by the bond between *HsfA2* and *HsfA1* (Heerklotz, Döring & Bonzelius, 2001). In *Arabidopsis*, *AtHsfA2* can be induced by HS. Basal and acquired thermotolerances, as well as the resistance to salt and the osmotic stresses can be enhanced in *AtHsfA2*-overexpressing *Arabidopsis* seedlings (Ogawa, Yamaguchi & Nishiuchi, 2007). Basal and acquired thermotolerances and antioxidant capacity are consistently reduced in mutant *athsfa2* (Li et al., 2005).

As a key regulator in the response to environmental stresses, AtHsfA2 plays an important role in the regulating response to multiple abiotic stresses including heat (Busch, Wunderlich & Schöffl, 2005; Schramm et al., 2006; Nishizawa et al., 2006; Charng et al., 2007). It can directly bind to HSEs in the promoter region of target genes or interact with AtHsfA1s to form a heterogenic complex and regulate the expression of downstream genes, particularly HSPs (Liu & Charng, 2013; Nishizawa-Yokoi et al., 2011). AtHsfA1s function as key regulators in *AtHsfA2* expression (Liu & Charng, 2013). AtHsfA1s and AtHsfA2 have distinct but overlapping functions in response to abiotic stresses (Liu & Charng, 2013; Nishizawa-Yokoi et al., 2011).

In maize, four HSF of A2 subclass, *ZmHsf01*, *ZmHsf04, ZmHsf05,* and *ZmHsf17*, have been identified (Lin et al., 2011). Ectopic overexpression of *ZmHsf04* and *ZmHsf05* improved the thermotolerance of transgenic *Arabidopsis* seedlings (Li et al., 2019; Jiang et al., 2018). In our study, we cloned and analyzed *ZmHsf01,* another member of A2 subclass, and observed the subcellular localization of ZmHsf01 protein, based on these, *ZmHsf01* functions in response to HS were detected and discussed further.

## Material and Methods

### Plant materials and culture conditions

Maize (*Zea mays* L.) inbred line H21 was used in this study. Healthy seeds were surface-sterilized with 0.1% HgCl_2_ for 10 min and rinsed repeatedly with double distilled water. The seeds germinated in the dark and were planted in a controlled environment greenhouse at 28 °C with conditions of 16/8 h of day/night (100 µmol m^−2^ s^−1^) and 60% relative humidity (RH). Seedlings with two leaves were used for stress experiments and young roots, shoots, and leaves were sampled, respectively. Mature leaves, pollen, and ears were separated during the blooming period. Immature embryos were separated two weeks after pollination. All tissues and organs were frozen in liquid nitrogen for gene expression analysis.

### Stress treatment

Uniform maize seedlings with two leaves were selected and subjected to treatment methods described by Li et al., with some modifications (Li et al., 2019). For HS treatment, the Hoagland nutrient solution was pre-heated at 42 °C before immersing into seedlings. Leaves and roots were sampled at treatment times of 0, 10, 20, 30, 40, 50, 60, and 120 min. For abscisic acid (ABA) treatment, the seedlings were treated with Hoagland nutrient solution with a concentration of 200 µM ABA. The treatment times were 0, 2, 4, 6, 12, 24, and 36 h. For H_2_O_2_ treatment, the seedlings were treated Hoagland nutrient solution with a final concentration of 10 mM H_2_O_2_. The treatment times were 0, 15, 30, 60, 90, 120, and 240 min. For qRT-PCR testing of HSPs, 5-day-old transgenic line and wild type (WT) seedlings were selected and the leaves both WT and overexpressing line were harvested at 2 h after HS treatment at 45 °C for 50 min for the basal thermotolerance and the the following treatment for the acquired thermotolerance: pre-treatment at 37 °C for 60 min, a recovery under the normal conditions for 2 days respectively, and then retreatment at 46 °C for 2 h. We carried out three biological experiments. The samples were collected and quickly frozen in liquid nitrogen.

### Gene cloning and sequencing

A total RNA extraction from leaves was conducted using the RNArose Reagent Systems Kit (SBS, Beijing, China). The genomic DNA was digested using *DNase* I (TaKaRa, Dalian, China) for 30 min at 37 °C. 1 µg total RNA was used for the first standard synthesis of cDNA using a Reverse Transcription Kit (Invitrogen, USA). The quantity of RNA samples was checked using a NanoDrop 2000 (Thermo Scientific, USA).

The primers (forward primer 5′-CGTGGCGAGATGGACCTGATGC-3′ and reverse primer 5′-TTAACGCGATCATCTCTACTTC-3′) were designed to amplify the open reading frame of *ZmHsf01*. We submitted the full *ZmHsf01* coding sequences (CDS) to GenBank under accession number MK888854. High-fidelity enzyme Pyrobest (TaKaRa, Dalian, China) was used for PCR amplification. The PCR reaction system consisted of 1 × Pyrobest buffer, 0.2 mM dNTP mixture, 200 ng first strand cDNA, 0.2 µM forward primer, 0.2 µM reverse primer, and 1.25 units Pyrobest DNA polymerase. The reaction procedure was 30 cycles of: 98 °C for 10 s, 55 °C for 15 s, and 72 °C for 2 min. The PCR products were ligated into T-Vector (TransGen Biotech, Beijing, China) and sequenced by the Shanghai Sangon Biotech Company.

### Quantitative real time PCR (qRT-PCR) analyses

The PCR reaction mixtures contained 1 × SYBR Premix Ex *Taq*II (Takara, Dalian, China), 0.4 µM forward primer, 0.4 µM reverse primer, and 1 µg cDNA for a final volume of 20 µL. We used a 7500 Real-Time PCR system (Applied Biosystems, USA) in this experiment. The reaction procedure was as follows: pre-denaturation at 95 ° C for 10 min, 40 cycles of denaturation at 95 °C for 5 s, and annealing/extension at 60 °C for 1 min. After the reaction, we analyzed the data using the 2^−ΔΔ*Ct*^ method. Three biological replicates were performed in each group of experiments. The data was analyzed with Microsoft Excel 2010. For the statistical analyses, each dataset was repeated at least three times. We set the expression level of young roots as 1 during the expression analysis of tissues and organs, and expression levels at 0 min were set as 1 for the expression analysis of different stress treatments. Maize gene *β*-Actin was used as an endogenous control, and *AtActin8* (At1g49240) was used for *Arabidopsis*. The primers of *ZmHsf01* and other relevant genes were listed in Table S1.

### Subcellular localization of *ZmHsf01* protein in tobacco epidermal cells

Using a ClonExpress II kit (Vazyme, Nanjing, China), we constructed a recombinant vector pCAMBIA1300-ZmHsf01-GFP driven by a CaMV 35S promoter containing the *ZmHsf01* CDS amplified by gene-specific primers (forward primer: 5′-GAGAACACGGGGGACTCTAGAATGGACCTGATGCTG-3′; reverse primer: 5′-GCCCTTGCTCACCATGGATCCCTTCGCCGTGGTGTT-3′) and the GFP gene. The constructs were transformed into *Agrobacterium tumefaciens* EHA105 competent cells by the freeze-thaw method. ZmHsf01-GFP was expressed in the epidermal cells of tobacco leaf using the infiltration method described by Runions, Hawes & Kurup (2007) with EHA105 cells. We raised the tobacco seedlings in a glasshouse (12/12 h of day/night, 150 µmol m - 2 s - 1 50% RH, 19−23 °C temperature) for 72 h, and harvested the leaves and stained them with DAPI (a nuclei-special dye) (10 µg mL^−1^) for 5 min. After being rinsed three times with physiological saline, the tobacco epidermal cells were observed under a laser-scanning confocal LSM 710 microscope (Zeiss Microsystems, Germany).

### Semi quantitative RT-PCR (semi qRT-PCR) assay

The transcription abundance of *ZmHsf01* in transgenic *Arabidopsis* was tested using semi qRT-PCR method. RNA extraction and cDNA synthesis were carried out according to the protocols mentioned above. Based on the coding region sequence of *ZmHsf01*, we synthesized a pair of primers (forward primer, 5′-GTGACGGTAAAGGAGGAGTGGCCT-3′; reverse primer, 5′-GCCATAGGTGTTCAGCTGGCGGAC-3′). *AtActin2* (At3g18780) (forward primers 5′-CAATCGTGTGTGACAATGG-3′ and reverse 5′-AACCCTCGTAGATTGGCA-3′) was used as a loading control.

### Construction and transformation of yeast expression vectors and thermotolerance assays

The pYES2 vector (Invitrogen, USA) was used to detect the target protein expression in *Saccharomyces cerevisiae*. Using the ClonExpress II recombination system (Vazyme, Nanjing, China), we amplified the PCR products of *ZmHsf01* CDS using a pair of specific primers (5′-GGGAATATTAAGCTTGGTACCATGGACCTGATGCTGCCG-3′and 5′- TGATGGATATCTGCAGAATTCCTACTTCGCCGTGGTGTT-3′) inserted into the pYES2 vector. The recombinants were transformed into INVSc1 competent yeast cells described by Gietz et al. (1992), and the cells were diluted and plated on a SC/Glu/Ura^−^ agar screening plate at 30 °C. After 2 to 3 days, the positive clones were selected and verified by colony PCR.

For the thermotolerance assays, the positive clones were cultured using a liquid SC/Glu/Ura^−^ medium in a shaking incubator (250 rpm min^−1^). When the OD_600_ of cells reached 0.6∼0.7, the cells were diluted to an OD_600_ of 0.2 with a SC/Glu/Ura^−^ liquid medium, and were cultured by shaking for 2∼3 h. Cells were collected at an OD_600_ of 0.4∼0.8, eluted twice with sterile water, and serially diluted to OD_600_ levels of 0.1, 0.05, and 0.01. Separated the two samples into two groups: one group subjected to HS treatment in a 50 °C water bath for 15 min followed by culturing at 30 °C, and the control group with no intervention. 8 µL treated yeast cells was plated on SC/Gal/Ura^−^ agar to be grown at 30 °C. Yeast colony formation was examined and photographed after 2∼3 days.

### Plasmid construction and transformation in *Arabidopsis*

Special primers (forward: 5′- GAGAACACGGGGGACTCTAGAATGGACCTGATGCTG-3′; reverse: 5′- CGATCGGGGAAATTCGAGCTCCTACTTCGCCGTGGTGTT -3′) were designed to amplify the coding sequence of *ZmHsf01* by RT-PCR. Using a ClonExpress II kit, we inserted the PCR products into the pCAMBIA1300 vector, digested in advance by *Xba I* and *Sac I* and driven by a CaMV 35S promoter. After the construct infected *Agrobacterium* GV3101, transformed them into the wild *Arabidopsis* and deletion mutants using the vacuum dipping method. The MS medium, containing 25 µg ml^−1^ hygromycin, was used to screen the progeny plants until the homozygous seeds were harvested. We used the transgenic T3 homozygous lines to identify thermotolerance.

### Thermotolerance assays in *Arabidopsis*

The sterilized WT, *athsfa2*, and transgenic line seeds of *Arabidopsis thaliana* (Ecotype, Columbia) were placed in 1/2 MS solid plates containing 0.8% agar. The plates were then placed in an incubator at 22 °C (during the day) and 18 °C (at night) with conditions of 16/8 h of day/night (100 µmol m^−2^ s^−1^). We evaluated the basal thermotolerance using the following protocol: 5-day-old seedlings of WT and three overexpressed lines were subjected to 45 °C HS for 50 min, then recovered under normal growth conditions for 8 days. The acquired thermotolerance was evaluated through following treatment: 5-day-old seedlings of WT and three overexpressed lines were subjected to 37 °C temperatures for 60 min, recovered under normal conditions for 2 h (short-time recovery) and 2 days (long-time recovery), respectively, then re-heated at 46 °C for 60 min, and recovered under normal conditions for 8 days. The complementary thermotolerance was assayed after 5-day-old WT, *athsfa2* mutant, and transgenic line seedlings were treated at 44 °C for 70 min and recovered under normal conditions for 8 days. We observed and photographed all phenotypes, measured the survival rates, and measured the chlorophyll content of the leaves (Li et al., 2015a). At least 30 seedlings for each line were analyzed, and all experiments were repeated for three times.

### Yeast transcriptional activation analysis

Transcription activation activity assays of ZmHsf01 was performed according to the Y2H system instructions (TaKaRa, Dalian, China). Using the CloneExpress II cloning kit, we created the recombinants with the pGBKT7 vector and the full *ZmHsf01* CDS amplified by specific primers (5′-ATGGCCATGGAGGCCGAATTCATGGACCTGATGCTG-3′ an 5′-CCGCTGCAGGTCGACGGATCCCTACTTCGCCGTGGT-3′). We used LiAc and PEG3350 in the yeast transformation assay. The pGBKT7, pGBKT7-53, and pGADT7-T vectors and the pGBKT7-ZmHsf01 fusion vectors were transformed into the yeast cell AH109. Transformed yeast cells of different concentrations were successively cultured in SD/Trp^−^ and SD/Trp^−^/His^−^/Ade^−^/X- α-gal for 5 days.

## Results

### Cloning of *ZmHsf01* and protein subcellular localization

**Figure 1:**
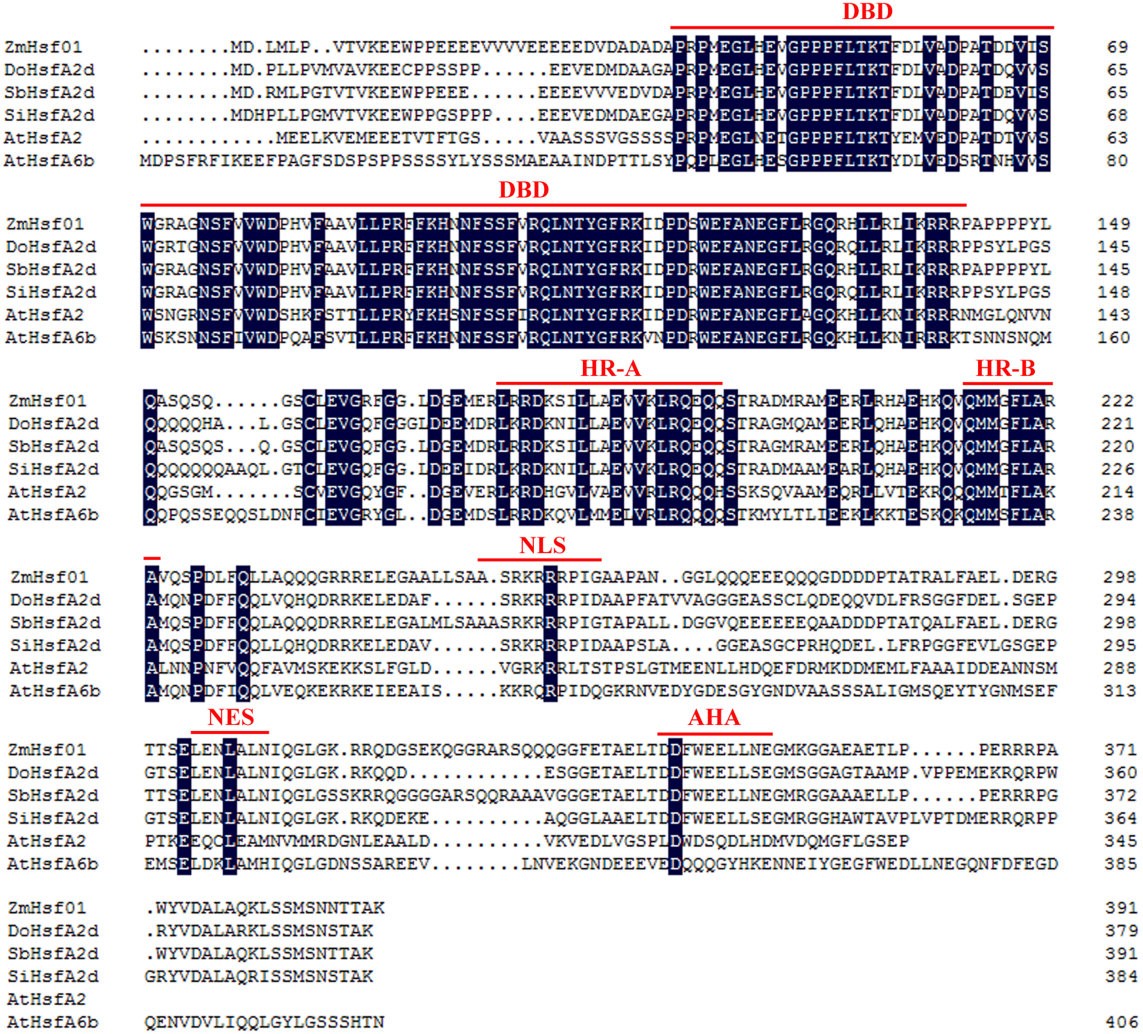
Amino acid sequence alignment between *ZmHsf01* and homologs from different species. DBD, the conserved DNA binding domain; HR-A/HR-B, two hydrophobic heptad repeats; NLS, nuclear localization signal; NES, nuclear export signal; AHA, aromatic, large hydrophobic and acidic amino residues. The protein accession numbers in NCBI are as follows: SbHsfA2d from *Sorghum bicolor*, XP_002468465.1; DoHsfA2d from *Dichanthelium oligosanthes*, OEL38242.1; SiHsfA2d from *Setaria italica*, XP_004985605.1; AtHsfA2 from *Arabidopsis thaliana*, AEC07800; and AtHsfA6b from *Arabidopsis thaliana*, AEE76681.

Using the RT-PCR method, we cloned the *ZmHsf01* CDS from H21 seedlings. Sequence analysis showed that the *ZmHsf01* CDS were 1,176 bp in length and encoded a deduced protein with 391 amino acid residues. The CDS and amino acid sequence are shown in Fig. S1. The conserved DNA-binding domain (DBD), oligomerization A/B (HR-A/B), nuclear localization signal (NLS), nuclear export signal (NES), and C-terminal activator motif (AHA) domains are marked with red lines (Fig. 1). Amino acid sequence alignment results showed that ZmHsf01 shared 86%, 70%, 69%, 42%, and 43% identities with SbHsfA2d from *sorghum bicolor* (XP_002468465), DoHsfA2d from *Dichanthelium oligosanthes* (OEL38242), SiHsfA2d from *Setaria italica* (XP_004985605), AtHsfA2 from *Arabidopsis thaliana* (AEC07800), and AtHsfA6b from *Arabidopsis thaliana* (AEE76681), respectively (Fig. 1). These results verify that *ZmHsf01* is a HSF gene of the A2 subclass.

**Figure 2:**
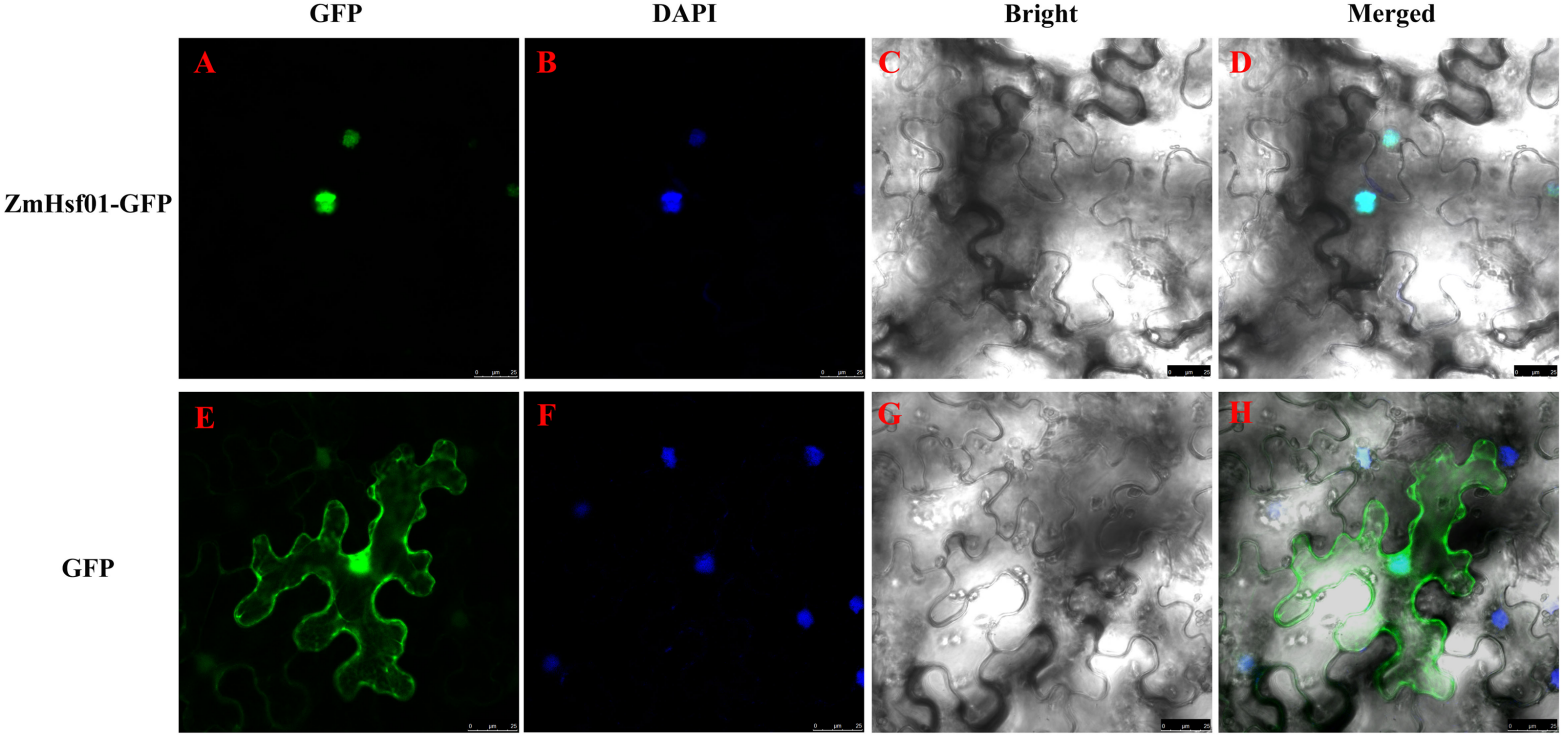
Subcellular localization of *ZmHsf01.* (A and E) Green fluorescence of GFP; (B and F) blue fluorescence of DAPI; (C and G) bright field; (D and H) merged images.

The ZmHsf01-GFP fusion protein were observed during the subcellular localization of ZmHsf01. The *ZmHsf01* CDS were connected to the N-terminal of the green fluorescent protein (GFP) gene driven by a CaMV 35S promoter. The tobacco epidermal cells expressed the ZmHsf01-GFP fusion protein by an *Agrobacterium*-mediated transformation. After cultured for 3 days, stained the tobacco epidermal cells with DAPI. The laser confocal microscopy examination found individual GFP proteins throughout the control group’s entire cell, but ZmHsf01-GFP fusion protein was only detected in the nuclei, and was co-localized using DAPI florescence (Fig. 2). These results suggest that ZmHsf01 is localized in the nucleus.

### Expression analysis of *ZmHsf01*

**Figure 3:**
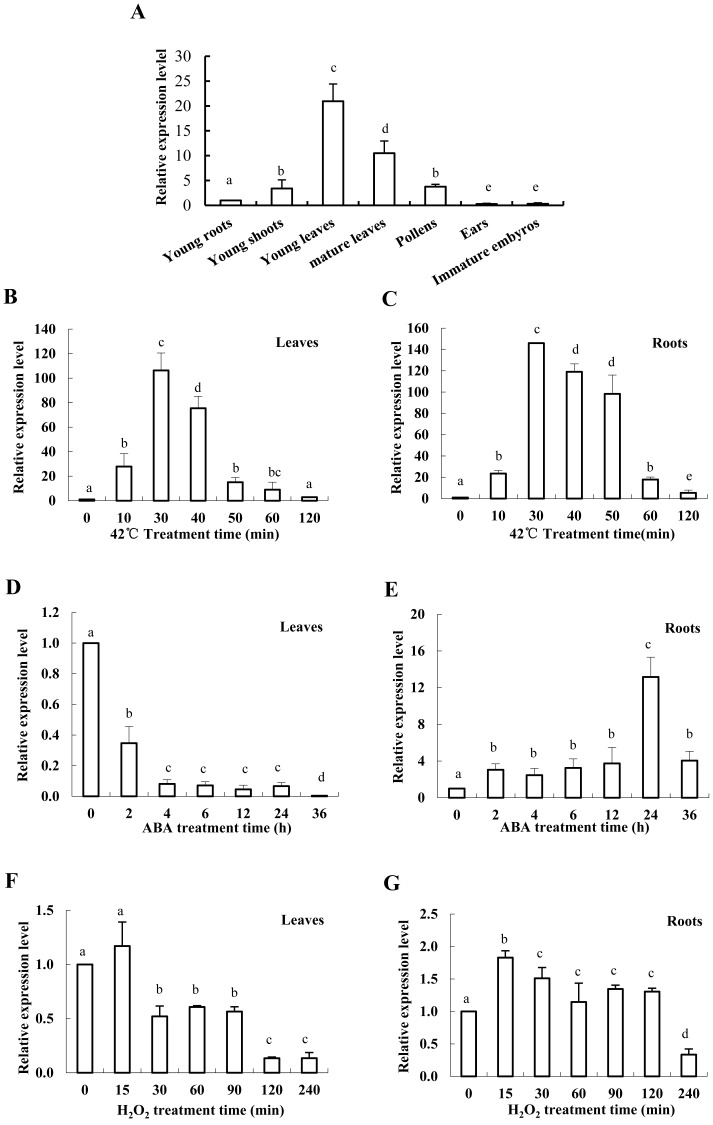
Expression patterns of *ZmHsf01* in different tissues and abiotic stresses include HS, ABA, and H_2_O _2_. (A) Expression levels of *ZmHsf01* in different tissues and organs under normal growth conditions. (B and C) Expression levels of *ZmHsf01* in leaves and roots of maize seedlings after 42 °C HS. (D and E) Expression levels of *ZmHsf01* in leaves and roots of maize seedlings after 200 μ M ABA treatment. (F and G) Expression levels of *ZmHsf01* in leaves and roots of maize seedlings after 10 mM H_2_O _2_ treatment. There were three biological repeats for each sample and the data mean ± standard error.

Under the normal growth conditions, *ZmHsf01* was expressed in all detected maize organs: young roots, young shoots, young leaves, mature leaves, pollen, ears, and immature embryos (Fig. 3A). The expression level of *ZmHsf01* was highest in young leaves and lowest in ears. *ZmHsf01* was expressed 20 times more in young leaves than in roots.

*ZmHsf01* expression was significantly up-regulated in both roots and leaves after 42 °C HS. Expression levels reached their peak at 30 min after HS, and then gradually decreased (Figs. 3B, 3C). After ABA treatment, the expression of *ZmHsf01* in roots was up-regulated and reached peak value at 24 h, but in leaves the *ZmHsf01* expression showed a tendency to be down-regulated (Fig. 3E). The expression level of *ZmHsf01* increased in roots and decreased in leaves under H_2_O_2_ treatment (Figs. 3F, 3G). These results suggest that *ZmHsf01* can be up-regulated by HS, ABA, and H_2_O _2_in roots(All the raw data of Fig. 3 were listed in Table S2).

### Improving thermotolerance through *ZmHsf01* expression in yeast cell

**Figure 4:**
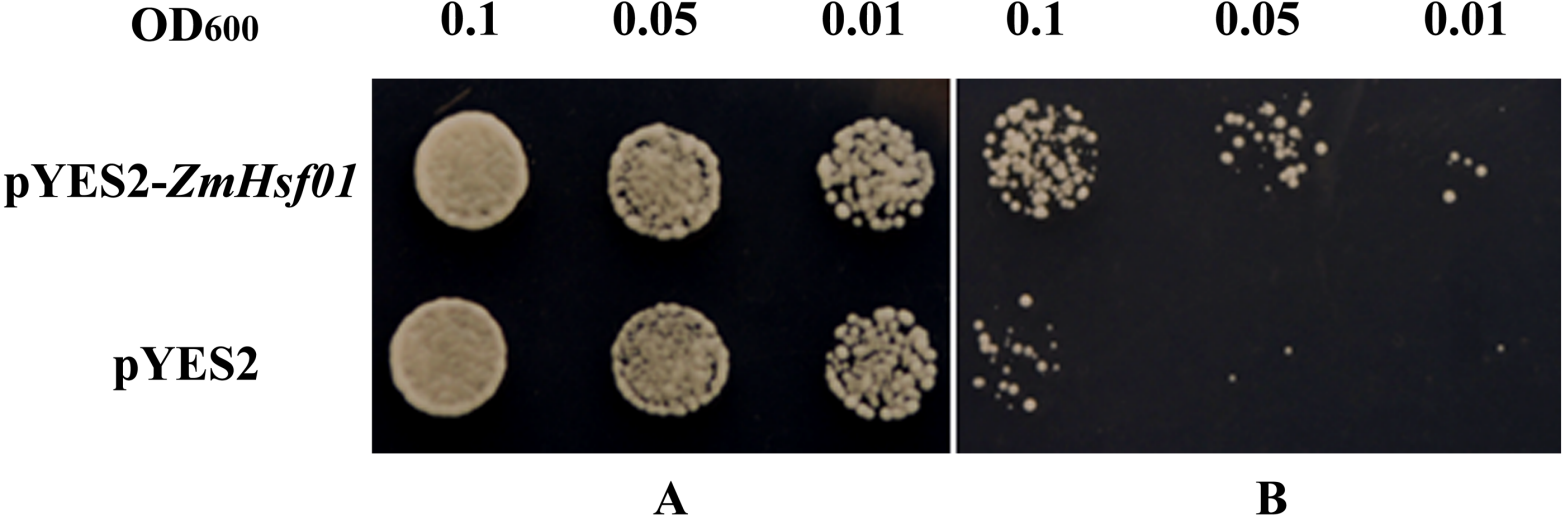
Thermotolerance assays of yeast cell harboring pYES2 or pYES2-ZmHsf01 after 50 °C HS. (A) Culture under normal conditions; (B) culture after HS at 50 °C for 15 min.

To further analyze the function of *ZmHsf0*1, we used the pYES2-ZmHsf01 yeast expression vector to identify the genetic transformation and thermotolerance of yeast positive strains. Under the normal conditions, no significant phenotype difference was found between the two types of transgenic yeast cells (pYES2-ZmHsf01 and pYES2 control). The cellular growth of the two groups was inhibited after heat treatment at 50 °C for 15 min, but the growth potential of *ZmHsf01*-expressing cells was better than that of the control cells (Fig. 4). These results demonstrate that *ZmHsf01* improves the thermotolerance of transgenic yeast cells.

### Yeast transcription activation activity of ZmHsf01

**Figure 5:**
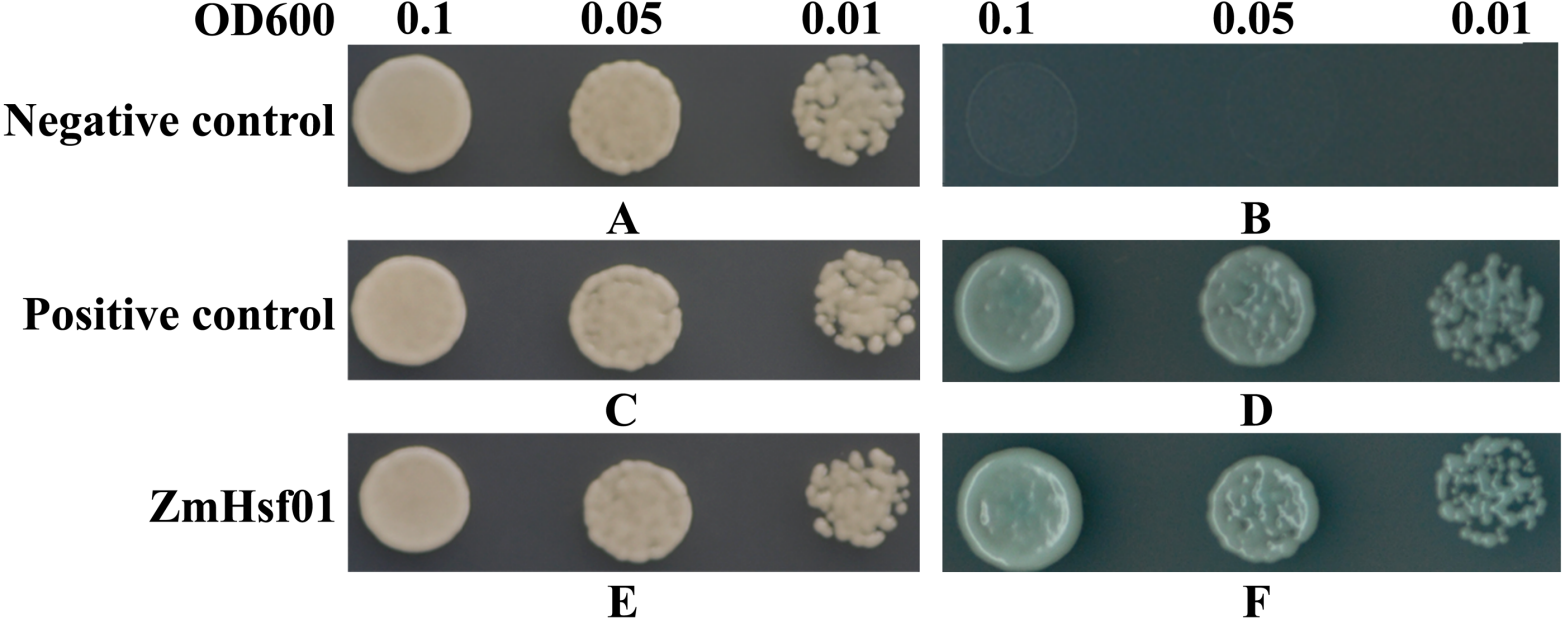
Transcription activation analysis of *ZmHsf01* in yeast. Yeast cells transformed with pGBKT7 vector were set as the negative control, and yeast cells co-transformed with pGBKT7-53 and pGADT7-T vectors were set as the positive control. The *ZmHsf01* group were the yeast cells transformed with the fusion vector pGBKT7-ZmHsf01. (A) The negative control in the SD/Trp^-^ culture medium. (B) The negative control in the SD/Trp^-^/His^-^/Ade^-^/X-α-gal culture medium. (C) The positive control in the SD/Trp^-^ culture medium. (D) The positive control in the SD/Trp^-^/His^-^/Ade^-^/X-α-gal culture medium. (E) The fusion vector pGBKT7-ZmHsf01 in the SD/Trp^-^ culture medium. (F) The fusion vector pGBKT7-ZmHsf01 in the SD/Trp^-^/His^-^/Ade^-^/X-α-gal culture medium.

Based on our domain analysis, ZmHsf01 contains the AHA domain existing in class A members. We constructed *ZmHsf01* into a pGBKT7 vector. The yeast strains transformed with fusion vector pGBKT7-ZmHsf01 grew well and turned blue similarly to the positive control groups in the SD/Trp-/His-/Ade-/X-α-gal culture medium (Fig. 5). Our results show that *ZmHsf01* is active in yeast cell transcription activation.

### *ZmHsf01* compensated for the thermotolerance defects of mutant *Arabidopsisathsfa2*

**Figure 6:**
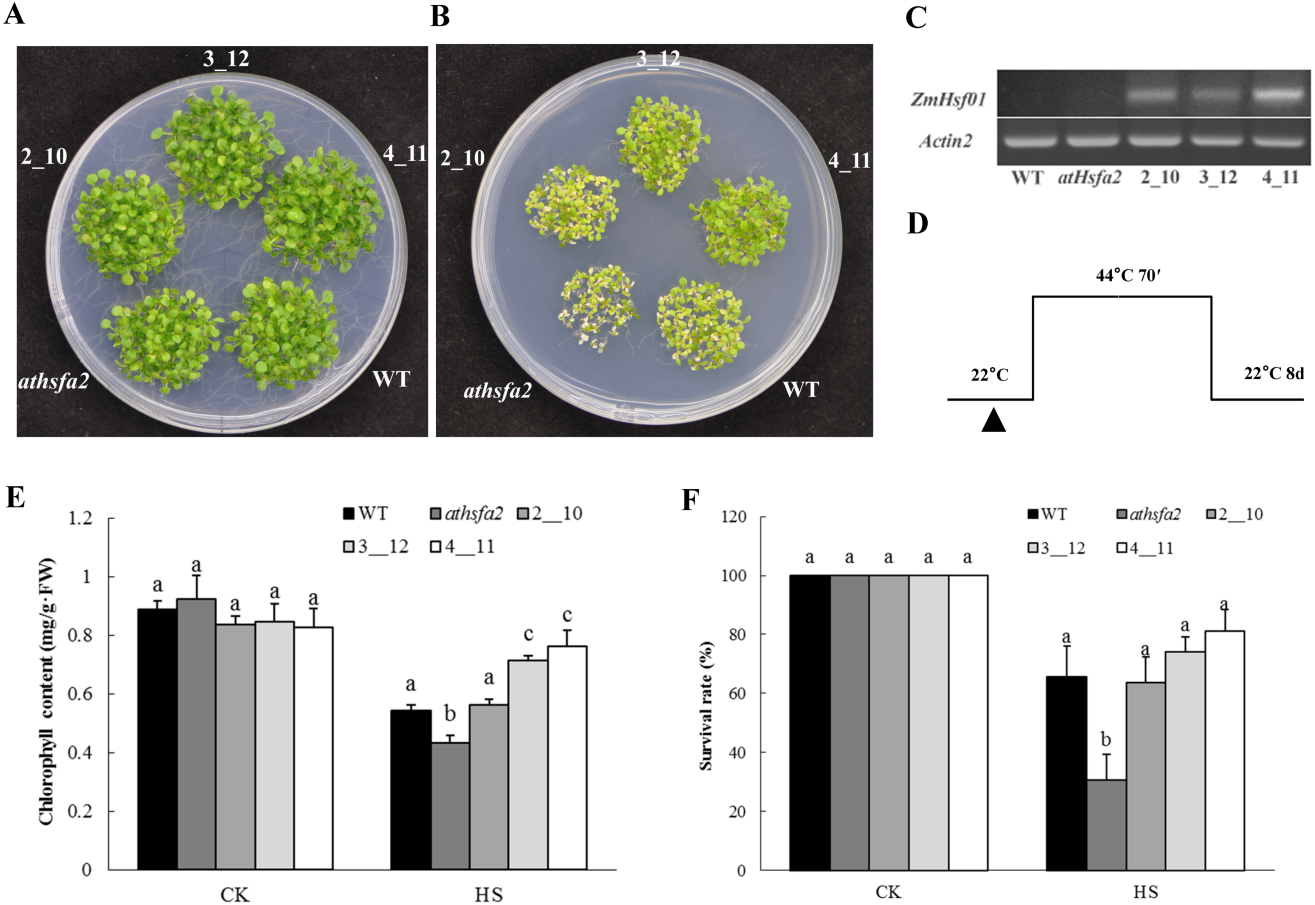
The thermotolerant phenotypes of the deletion mutant and three restoration lines of *Arabidopsis* seedlings. (A) Seedlings of WT, deletion mutant, and three restoration lines (2_10, 3_12, and 4_11) growing on the MS plate under normal conditions (control). (B) Seedlings of all genotypes under HS at 44 °C for 70 min and recovered under normal conditions for 8 days. (C) Chlorophyll content of seedlings under normal conditions and HS treatments. (D) Semi-qRT-PCR assay of the *ZmHsf01* transcript. (E) Schematic representation of the HS regimes.

Charng et al. (2007) first reported the thermotolerance defects of *Arabidopsis* mutant *athsfa2* (SALK_008978). We used the mutant *athsfa2* to investigate the thermotolerance of *ZmHsf01*. Three *ZmHsf01*/*athsfa2*-complemented lines (2_10, 3_1, and 4_11) that expressed different levels of *ZmHsf01* were selected using semi-quantitative RT-PCR (Fig. 6C). The seedlings of the three *ZmHsf01*/*athsfa2*-complemented lines, *athsfa2* and WT were photographed, and the chlorophyll content were measured under normal conditions and HS (Fig. 6). The thermotolerances of *ZmHsf01*/*athsfa2* were better than that both *athsfa2* and WT (Fig. 6B). The chlorophyll contents of *ZmHsf01*/*athsfa2* in lines 3_12 and 4_11 were higher than those of *athsfa2* and WT (Fig. 6E). The survival rates of all complemented lines were higher than the mutant *athsfa2*, but not WT (Fig. 6F). The results showed that *ZmHsf01* can partially or completely compensate for the thermotolerance defects of *athsfa2*.

### Functionally identifying *ZmHsf01* thermotolerance in *Arabidopsis*

**Figure 7:**
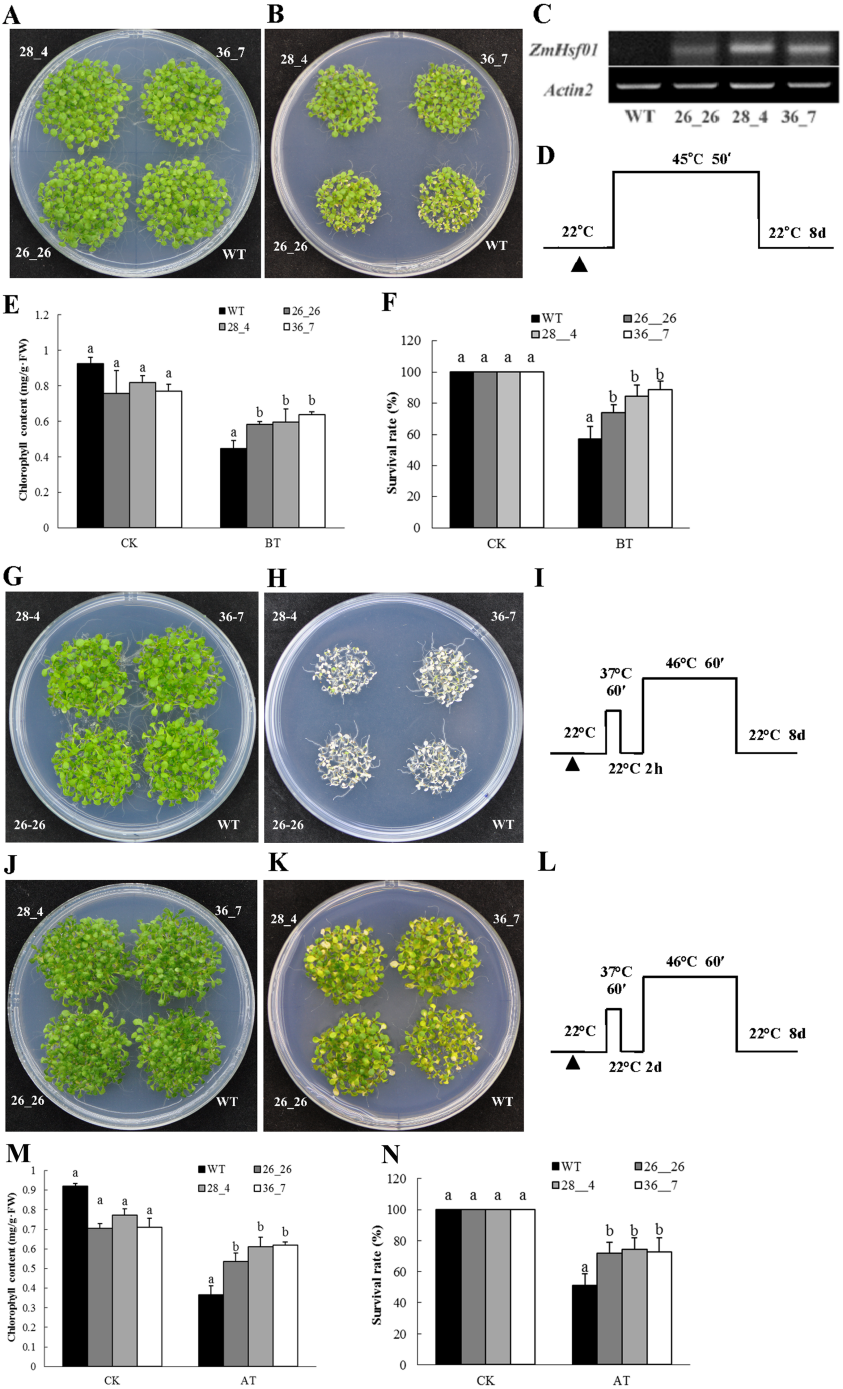
Overexpression of *ZmHsf01* improved the basal and acquired thermotolerance of *Arabidopsis* seedlings. (A) Seedlings of WT and three overexpressing lines (26_26, 28_4, and 36_7) growing on an MS plate under normal conditions used as the basal thermotolerance control. (B) Seedlings of all genotypes under basal HS at 45 °C for 50 min and recovered under normal conditions for 8 days. (C) Semi-qRT-PCR analysis of the *ZmHsf01* transcript in different lines. The expression levels of *AtActin2* were used as the control. (D) Schematic representation of the basal HS regimes. (E) Chlorophyll content of seedlings under normal conditions and basal HS treatments. (F) The survival rates of seedlings under normal conditions and basal HS treatments. (G) Seedlings of WT and three overexpressing lines (26_26, 28_4, and 36_7) growing on an MS plate under normal conditions used as the acquired thermotolerance control. (H) 5-day-old seedlings of all genotypes under acquired HS at 37 °C for 60 min, recovered under normal conditions for 2 h, treated at 46 °C for 60 min, and recovered under normal conditions for 8 days. (I) Schematic representation of the acquired HS regimes. (J) Seedlings of WT and three overexpressing lines (26_26, 28_4, and 36_7) growing on an MS plate under normal conditions used as the acquired thermotolerance control. (K) 5-day-old seedlings of all genotypes under acquired HS at 37 °C for 60 min, recovered under normal conditions for 2 days, treated at 46 °C C for 60 min, and recovered under normal conditions for 8 days. (L) Schematic representation of the acquired HS regimes. (M) Chlorophyll content of seedlings under normal conditions and acquired HS treatments. (N) The survival rates of seedlings under normal conditions and acquired HS treatments.

Three *ZmHsf01*-overexpressing lines (26_26, 28_4, and 36_7) were used to analyze the basal and acquired thermotolerances with different expression levels detected by semi-quantitative RT-PCR (Fig. 7C). Five-day-old seedlings of WT and three *ZmHsf01*-overexpressing lines growing on the same MS medium were exposed to special HS regimes (Fig. 7B), then recovered at 22 °C for 8 days (Fig. 7H). Under the normal conditions, no obvious phenotypic changes could be observed between the *ZmHsf01*-overexpressing lines and WT (Figs. 7A, 7G and 7J). After basal HS treatment and acquired HS treatment with a long-time recovery, the WT seedlings wilted, but the three *ZmHsf01* over-expressing lines remained green (Figs. 7B and 7K). However, after acquired HS treatment with a short-time recovery, the seedlings of WT and three *ZmHsf01* over-expressing lines all died (Fig. 7H). Further, we measured the chlorophyll contents and survival rates of different lines. Without HS treatment, no remarkable difference in chlorophyll content was found between the WT and three transgenic lines (Figs. 7E and 7M). After basal and acquired HS treatment, the chlorophyll contents of all genotypes decreased, but the chlorophyll contents of the three *ZmHsf01*-overexpressing lines were higher than that of WT (Figs. 7E and 7M), and the survival rates of the three over-expressing lines were higher than WT (Figs. 7F and 7N).

### Affection of overexpression of ZmHsf01 in *Arabidopsis* on AtHsps expression

**Figure 8:**
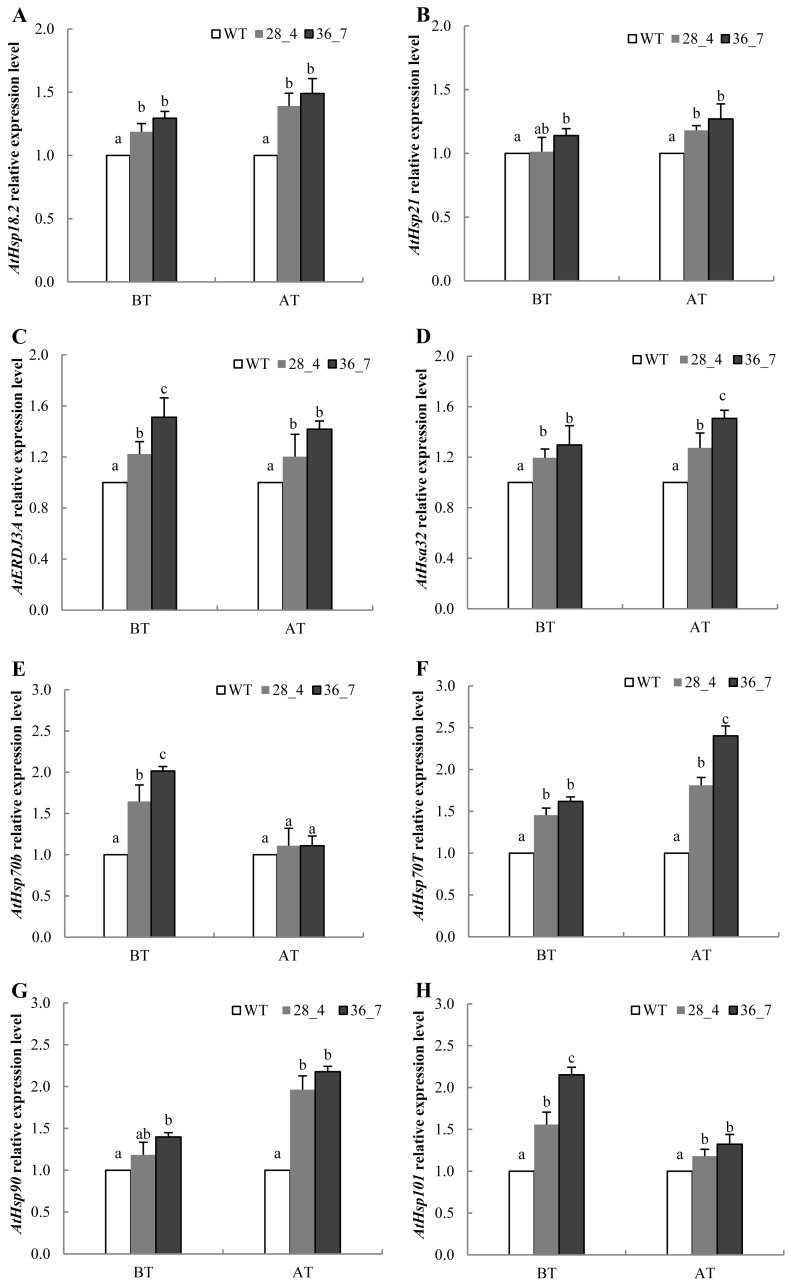
The expression levels of heat-related HSP genes in WT and *ZmHsf01* over-expressing lines 28_4 and 36_7 after HS treatment. qRT-PCR was performed on the *Arabidopsis* genes *AtHsp18.2* (A), *AtHsp21* (B), *AtERDJ3A* (C), *AtHsfa32* (D), *AtHsp70b* (E), *AtHsp70T* (F), *AtHsp90* (G), and *AtHsp101* (H) under BT and AT HS. We set the expression level of WT samples as 1. The reference gene *Atactin8* (At1g49240) was used as an internal control to normalize the loading of different samples. Data means ± SD from three biological experiments.

It has been shown that HSP genes can be induced and accumulate in cells so as to enhance the resistance of *Arabidopsis* plants under HS (Wan et al., 2016). To test the regulating role of *ZmHsf01* in HSPs expression, we performed qRT-PCR using *ZmHsf01* over-expressing lines 28_4 and 36_7. Five-day-old seedlings of WT and three *ZmHsf01*-overexpressing lines growing on the same MS medium were exposed to the HS treatment described in the Methods section. Such *AtHsps*, including *AtHsp18.2*, *AtHsp21*, *AtHsa32*, *AtERDJ3A*, *AtHsp70b*, *AtHsp70T*, *AtHsp90.1,* and *AtHsp101*, were detected after the basal and acquired heat treatments (Fig. 8). The results showed that the expression levels of *AtHsps* in *ZmHsf01*-overexpressing line 36_7 were 1.1 to 2.5 times higher than that in WT after HS treatment. The up-regulation of AtHsp70b and AtHsp101 only existed in BT, and the up-regulation of AtHsp70T and AtHsp90 was more obvious in AT. These results indicate that *ZmHsf01* can increase the expression of *AtHsps* to enhance the thermotolerance of transgenic *Arabidopsis* (The raw data were listed in Table S3).

## Discussion

In the HSF studies of *Arabidopsis* and tomato, the A1 and A2 subclasses were treated as the main research focus (Nakai, 2016; Scharf et al., 1998; Ogawa, Yamaguchi & Nishiuchi, 2007; Liu & Charng, 2013). With the recent developing in crop genome sequencing, HSF family members in more and more species have been speculated and genetically analyzed. The functional analysis of other subclass members have also been gradually developed (Ma et al., 2016; Hu et al., 2018). Research on HSFs in field crops started relatively late. Many transcription factor (TF) families, such as WRKY, MYB, DREB, and HSF, have enormous potential for improving the resistance of maize (Kimotho, Baillo & Zhang, 2019). In 2011, 25 HSFs were identified in maize, and total 30 *ZmHsfs* in 2016 (Lin et al., 2011; Guo et al., 2016). Xue et al. reported that at least 56 HSFs exist in wheat (Xue et al., 2014), but our analysis shows a more larger number family (Duan et al., 2019), suggesting the complexity and diversity of HSF family.

In *Arabidopsis*, A1 subclass HSFs are considered the master regulators in sensing stress and downstream gene activation (Liu et al., 2013). Unlike A1 subclass, the genes of A2 subclass are always induced after HS (Schramm et al., 2006; Mittal et al., 2009). *ZmHsf01* expression after 30 min of HS increased and then decreased in leaves and roots. ZmHsf01 contains the typical domains of the HsfA class, such as DBD, HR-A/HR-B, NLS, and AHA motifs. ZmHsf01-GFP fusion protein is localized in the nuclei, which is consistent with the structure of the NLS motif within *ZmHsf01* protein. This expression level trend in *ZmHsf01* corresponds with previously reported ZmHsfA2s (Li et al., 2019; Jiang et al., 2018). The conserved domain among these class A members suggests similarities among gene functions. ABA, low temperatures, and NaCl treatments significantly induced the up-regulation of *ZmHsf04* (Li et al., 2019). However, *ZmHsf01* was only up-regulated after ABA or H_2_O_2_ treatment in the roots, and the *ZmHsf01* expression levels were lower in leaves. It has been known that ABA can regulate tolerance to various abiotic stress, and exogenous ABA could induce the expression of HsfA2 and enhance the heat tolerance of *Arabidopsis* and tall fescue (Wang et al., 2017b). However, the expression levels of *SbHsfA2* genes in sorghum leaves remained stable after 100 µM of ABA for 4 h (Nagaraju et al., 2015). H_2_O_2_ was also involved in the regulation of some stress responses. The *ZmHsf06* expression could be up-regulated by 42 °C HS and H_2_O_2_, and this expression was H_2_O_2_-dependent (Li, Li & Guo, 2015b). In the process of acquired thermotolerance, the regulation of salicylic acid (SA) on the *AtHsfA2* also depend on the existence of H_2_O_2_ signaling pathway(Gaffney et al., 1993; Snyman & Cronje, 2008). ABA up-regulated the *OsHsfA2* and *OsHsfA4a* expression by inducing H_2_O_2_ in rice (Dang, Jiang & Lin, 2010), and the up-regulation of ABA on *ZmHsf06* also partly depended on H_2_O_2_ (Li, Li & Guo, 2015b). The expression of *OsHsfA2d* increased 4∼6 folds under salt, PEG, and cold stress (Liu et al., 2010). The abiotic stress assays suggest that *HsfA2s* are induced by various environmental stresses, but showed different response pattern.

Previous studies on *Arabidopsis* have shown that AtHsfA2 can sustain the expression of HSP genes during the recovery stage and the acquired thermotolerance (Charng et al., 2007). There is only one *HsfA2* in *Arabidopsis*, compared with WT, mutant *athsfa2* is more sensitive to HS (Li et al., 2005; Charng et al., 2007). *FaHsfA2c* from tall fescue can improve the heat tolerance of mutant *Arabidopsis athsfa2* (Wang et al., 2017a). *ZmHsf05*, another member of class A2, can also complement the lack of thermotolerance of mutant *athsfa2* mutant (Li et al., 2019). In this study, *ZmHsf01* rescued the thermotolerant phenotypes of the *athsfa2* mutant, and *ZmHsf01*-overexpressing *Arabidopsis* seedlings improved the basal and acquired thermotolerance when compared with WT. These results demonstrate that *ZmHsf01* can improve thermotolerance, playing a similar role to *ZmHsf05*. However, the other roles of *ZmHsf01* such as involving in salinity and drought stresses have not been reported, it’s necessary to be studied further. Wheat *TaHsfA2d* has been proved to improve tolerance to high temperatures, salinity, and drought stresses in transgenic *Arabidopsis* plants (Chauhan et al., 2013).

Working as molecular chaperones, HSPs belong to multigene families and participate in various biological processes: protein folding, refolding, co-degradation of denatured proteins, and normal growth development (Kuang et al., 2017; Queitsch et al., 2000). The recognized “refolding machines”, Hsp70 and ERDJ3A, can refold denatured proteins under HS and alleviate stress damage (Ma et al., 2015). Both the regulation of HSFs on HSPs and the feedback inhibition of HSPs on HSFs play vital role in stress regulation (Frangkostefanakis et al., 2015). In both *ZmHsf04* and *Zmhsf05* over-expressing *Arabidopsis*, the expression levels of all detected AtHsps were higher than those in WT after HS (Li et al., 2019; Jiang et al., 2018). After heat treatment, the transcript levels of some HSPs, such as AtHsp70b, AtHsp70T, AtHsp90, and AtHsp101, in *ZmHsf01* over-expressing lines were higher than those of WT. At the same time, the up-regulation of AtHsp70b and AtHsp101 only existed in BT, while the up-regulation of AtHsp70T and AtHsp90 was more obvious in AT. These results indicate that *ZmHsf01* may improve the different thermotolerance of plants by regulating different HSPs expression.

Our analysis proved that heat treatment may help plants accumulate various HSPs to improve their thermotolerance. Different member of the HSF family plays different role in HS signal transduction and downstream gene expression. Further tests of the interactions of different HSFs and the gene regulatory mechanism in transgenic maize should be conducted.

## Conclusion

We cloned *ZmHsf01* from maize inbred line H21. *ZmHsf01* was highly conserved compared to its homologs in other plants. *ZmHsf01* compensated for the thermotolerant defects of *athsfa2* mutant of *Arabidopsis thaliana*. The *Arabidopsis* seedlings of overexpressing *ZmHsf01* had more stronger thermotolerance than WT seedlings.

##  Supplemental Information

10.7717/peerj.8926/supp-1 Figure S1 The CDS and amino acid sequences of *ZmHsf01*

10.7717/peerj.8926/supp-2 Table S1 The qPCR primers of *Zea mays* L. and *Arabidopsis thaliana* L. ** The accession numbers of cDNA sequences of genes from *Arabidopsis* available at NCBI. The forward and reverse primers were designed with the software Primer Premier 5.

10.7717/peerj.8926/supp-3 Table S2 The raw data of expression patterns of ZmHsf01 in tissues and under abiotic stresses Abiotic stresses include heat stress, ABA, and H_2_O_2_. A, Expression levels of ZmHsf01 in different tissues and organs under normal growth conditions. B and C, Expression levels of ZmHsf01 in leaves and roots of maize seedlings after 42 °C heat shock. D and E, Expression levels of ZmHsf01 in leaves and roots of maize seedlings after 200μ M ABA treatment. F and G, Expression levels of ZmHsf01 in leaves and roots of maize seedlings after 10 mM H_2_O_2_ treatment. There were three biological repeats for each sample and the data mean ± standard error.

10.7717/peerj.8926/supp-4 Table S3 The raw data of expression levels of heat-related Hsp genes in WT and ZmHsf01 over-expressing lines after HS treatment qRT-PCR was performed on the *Arabidopsis* AtHsp18.2, AtHsp21, AtERDJ3A, AtHsfa32, AtHsp70b, AtHsp70T, AtHsp90, and AtHsp101 under BT and AT HS. We set the expression level of WT samples as 1. The reference gene Atactin8 (At1g49240) was used as an internal control to normalize the loading of different samples. Data means ± SD from three biological experiments.

10.7717/peerj.8926/supp-5 Supplemental Information 1 The uncropped blots of Arabidopsis β-actin in Fig. 6C The β-actin blots of WT, athsfa2 and three over-expressing lines are tagged with white words.

10.7717/peerj.8926/supp-6 Supplemental Information 2 The uncropped blots of ZmHsf01 in Fig. 6C The ZmHsf01 blots of WT, athsfa2 and three over-expressing lines are tagged with white words.

10.7717/peerj.8926/supp-7 Supplemental Information 3 The uncropped blots of Arabidopsis β-actin in Fig. 7C The β-actin blots of WT and three over-expressing lines are tagged with white words.

10.7717/peerj.8926/supp-8 Supplemental Information 4 The uncropped blots of ZmHsf01 in Fig. 7C The ZmHsf01 blots of WT and three over-expressing lines are tagged with white words.

## Abbreviations

HSFs: heat shock transcription factors
HSE: heat shock element
HS: heat shock
HSPs: heat shock proteins
RH: relative humidity
CDS: coding sequence
DBD: DNA-binding domain
OD: oligomerization domain
NLS: nuclear localization signal
NES: nuclear export signal
AHA motifs: C-terminal activator motifs
ABA: abscisic acid
SA: salicylic acid
H_2_O_2_: hydrogen peroxide
PEG: polyethylene glycol
GFP: green fluorescence protein
WT: wild type
BT: basal thermotolerance
AT: acquired thermotolerance

## Additional Information and Declarations

### Data Availability

The following information was supplied regarding data availability: The raw measurements are available in the Supplemental Files and the ZmHsf01 CDS and amino acid sequences are available at GenBank: MK888854.
