# Supplementary material for: Functional characterization of maize heat shock transcription factor gene ZmHsf01 in thermotolerance
Source: PeerJ. 2020 Apr 10;8:e8926. doi: 10.7717/peerj.8926 (PMC7153558; doi:10.7717/peerj.8926)
Supplement: Figure S1 [file peerj-08-8926-s001.pdf]

```
1        ATGGACCTGATGCTGCCGGTGACGGTAAAGGAGGAGTGGCCTCCGGAGGAGGAGGAGGTG
1         M  D  L  M  L  P  V  T  V  K  E  E  W  P  P  E  E  E  E  V

61       GTGGTGGTGGAGGAGGAGGAGGAGGACGTGGACGCGGACGCGGACGCTCCGCGGCCGATG
21        V  V  V  E  E  E  E  E  D  V  D  A  D  A  D  A  P  R  P  M

121      GAGGGGCTGCACGAGGTCGGGCCACCGCCGTTCCTGACCAAGACGTTCGACCTGGTGGCC
41        E  G  L  H  E  V  G  P  P  P  F  L  T  K  T  F  D  L  V  A

181      GACCCGGCCACCGACGACGTCATCTCCTGGGGCCGCGCCGGCAACAGCTTCGTGGTCTGG
61        D  P  A  T  D  D  V  I  S  W  G  R  A  G  N  S  F  V  V  W

241      GATCCCCACGTGTTCGCCGCCGTGCTGCTCCCCAGGTTCTTTAAGCACAACAACTTCTCC
81        D  P  H  V  F  A  A  V  L  L  P  R  F  F  K  H  N  N  F  S

301      AGCTTTGTCCGCCAGCTGAACACCTATGGCTTCAGAAAGATCGACCCGGACAGCTGGGAG
101       S  F  V  R  Q  L  N  T  Y  G  F  R  K  I  D  P  D  S  W  E

361      TTCGCGAACGAAGGATTCCTGAGGGGCCAGAGGCATCTTCTCCGGCTGATCAAGCGTCGG
121       F  A  N  E  G  F  L  R  G  Q  R  H  L  L  R  L  I  K  R  R

421      AGGCCGGCGCCGCCGCCGCCGTACCTCCAGGCGTCGCAGTCGCAGGGGTCGTGCCTGGAG
141       R  P  A  P  P  P  P  Y  L  Q  A  S  Q  S  Q  G  S  C  L  E

481      GTGGGCCGGTTCGGGGGGCTGGACGGAGAGATGGAGCGGCTGAGGCGCGACAAAAGCATC
161       V  G  R  F  G  G  L  D  G  E  M  E  R  L  R  R  D  K  S  I

541      CTGCTGGCGGAGGTGGTGAAGCTGCGGCAGGAGCAGCAGAGCACGCGGGCGGACATGCGG
181       L  L  A  E  V  V  K  L  R  Q  E  Q  Q  S  T  R  A  D  M  R

601      GCCATGGAGGAGCGGCTGCGGCACGCGGAGCACAAGCAGGTGCAGATGATGGGGTTCCTG
201       A  M  E  E  R  L  R  H  A  E  H  K  Q  V  Q  M  M  G  F  L

661      GCGCGGGCGGTGCAGAGCCCGGACTTGTTCCAGCTGCTGGCCCAGCAGCAGGGCAGGCGG
221       A  R  A  V  Q  S  P  D  L  F  Q  L  L  A  Q  Q  Q  G  R  R

721      AGGGAGCTGGAGGGCGCGGCGCTGCTCTCCGCCGCCTCCCGGAAGCGGAGGCGGCCCATC
241       R  E  L  E  G  A  A  L  L  S  A  A  S  R  K  R  R  R  P  I

781      GGCGCCGCGCCGGCCAACGGCGGCTTGCAGCAGCAGGAGGAGGAGCAGCAGCAGGGCGAC
261       G  A  A  P  A  N  G  G  L  Q  Q  Q  E  E  E  Q  Q  Q  G  D

841      GACGACGACCCCACCGCCACGCGGGCGCTGTTCGCGGAGCTGGACGAGCGAGGGACCACG
281       D  D  D  P  T  A  T  R  A  L  F  A  E  L  D  E  R  G  T  T

901      TCGGAGCTGGAGAACCTGGCGCTCAACATCCAGGGGCTCGGCAAGCGCAGGCAGGACGGG
301       S  E  L  E  N  L  A  L  N  I  Q  G  L  G  K  R  R  Q  D  G

961      AGCGAGAAGCAGGGTGGCCGCGCGCGGAGCCAGCAGCAGGGCGGGTTCGAGACGGCGGAG
321       S  E  K  Q  G  G  R  A  R  S  Q  Q  Q  G  G  F  E  T  A  E

1021     CTCACCGACGACTTTTGGGAGGAGCTGCTGAACGAAGGGATGAAGGGCGGTGCCGAGGCT
341       L  T  D  D  F  W  E  E  L  L  N  E  G  M  K  G  G  A  E  A

1081     GAGACGCTGCCGCCGGAGAGGAGACGACCGGCTTGGTACGTCGACGCGCTGGCGCAGAAG
361       E  T  L  P  P  E  R  R  R  P  A  W  Y  V  D  A  L  A  Q  K

1141     TTGAGCTCCATGAGCAATAACACCACGGCGAAGTAG
381       L  S  S  M  S  N  N  T  T  A  K  *
```
