# Supplementary material for: Functional characterization of maize heat shock transcription factor gene ZmHsf01 in thermotolerance
Source: PeerJ. 2020 Apr 10;8:e8926. doi: 10.7717/peerj.8926 (PMC7153558; doi:10.7717/peerj.8926)
Supplement: Table S1 — The accession numbers of cDNA sequences of genes from Arabidopsis available at NCBI. The forward and reverse primers were designed with the software Primer Premier 5. [file peerj-08-8926-s002.pdf]

Gene Name	Accession number	Forward	Reverse
ZmHsf01	MK888854	AGAACCTGGCGCTCAACA	TCAGCAGCTCCTCCCAAA
AtHsp18.2	At5g59720	GCAGATTAGCGGAGAGAGGA	CCTTCACTTCTTCCATCTTTGC
AtHsp21	At4g27670	AAGTCCGCTACACCGTTCTC	CCAACAATCCGAAAGGAGAG
AtERDJ3A	At3g08970	CTCCTGTTTGTATCATTGGTGC	TGTGTCCTGAGAACCTGTGG
AtHsa32	At4g21320	GCGAAGTTGGTTGAGTGGTT	GGAGGAACTGAGAACAGATTGG
AtHsp70b	At1g16030	TCCGCTTAGCCTTGGACTT	ACGCCTGGTTGATTGTCTG
AtHsp70T	At2g32120	TGATTGAGGTGAGGATGCC	CCACTTCAACGACAAACCC
AtHsp90.1	At5g52640	CCCTCTCTTCTTCATAAATCAACA	CCATCGCAACGAACTTTG
AtHsp101	At1g74310	TGTCTTCAACACTCTGCTCCA	CACTTCCATTGTTACTTTCCCAG
AtActin8	At1g49240	CTCTCAATCGCATACACCAGC	ATCCACTAAGCACTTGCCTCA
