# Supplementary figures and images for: Functional characterization of maize heat shock transcription factor gene ZmHsf01 in thermotolerance
Source: PeerJ. 2020 Apr 10;8:e8926. doi: 10.7717/peerj.8926 (PMC7153558; doi:10.7717/peerj.8926)

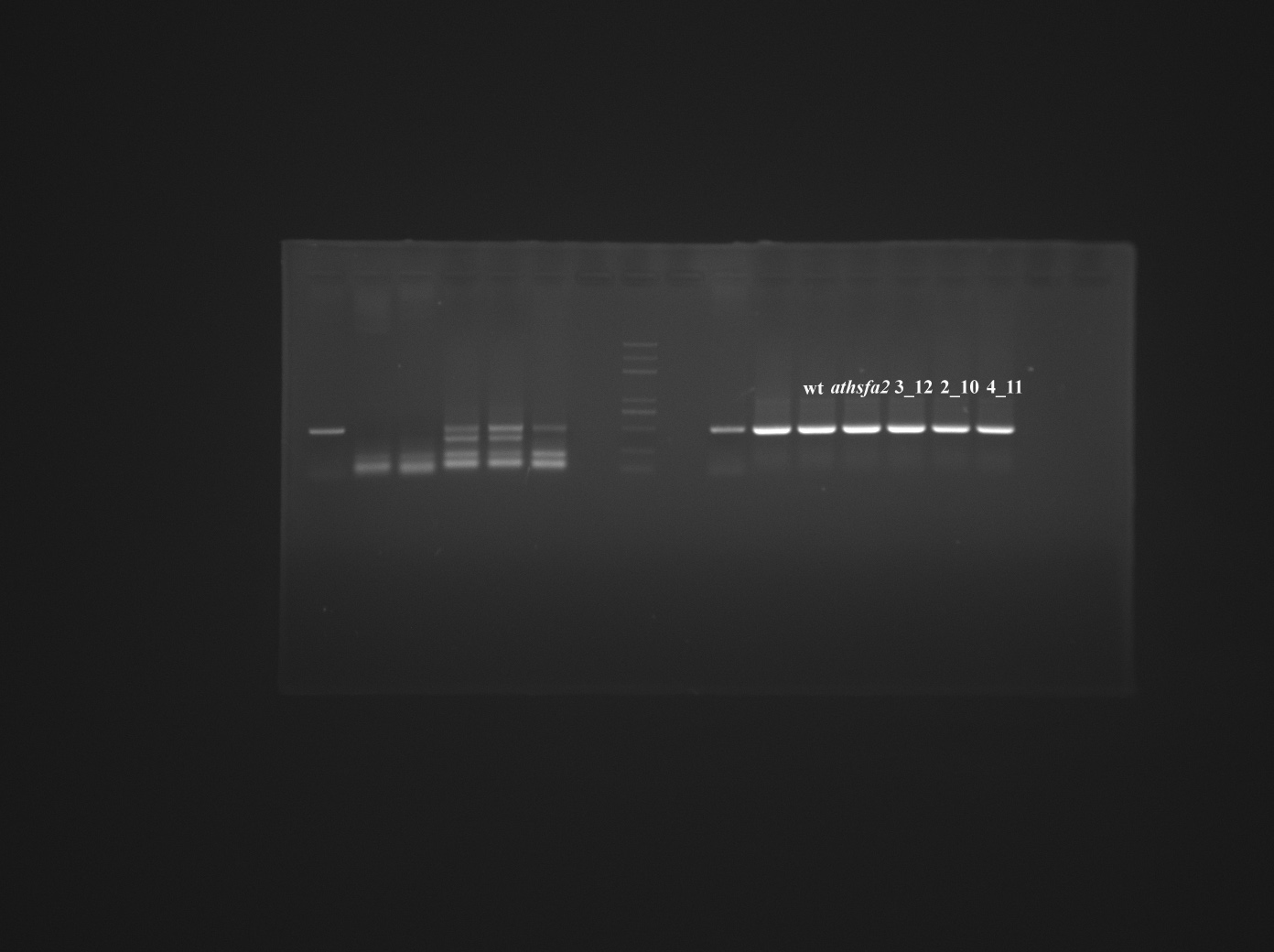

Supplement: Supplemental Information 1 — The β-actin blots of WT, athsfa2 and three over-expressing lines are tagged with white words. [file peerj-08-8926-s005.jpg]

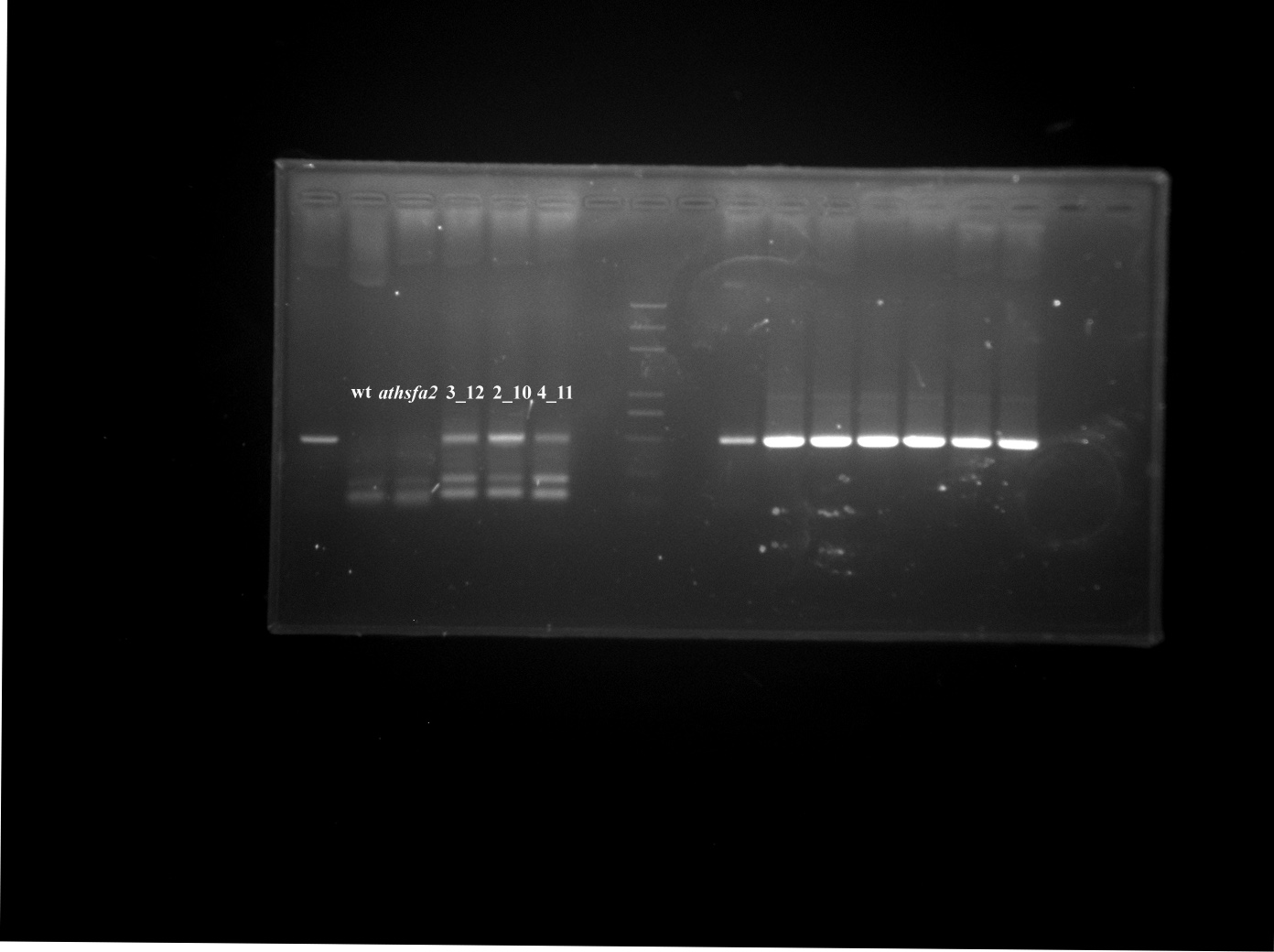

Supplement: Supplemental Information 2 — The ZmHsf01 blots of WT, athsfa2 and three over-expressing lines are tagged with white words. [file peerj-08-8926-s006.jpg]

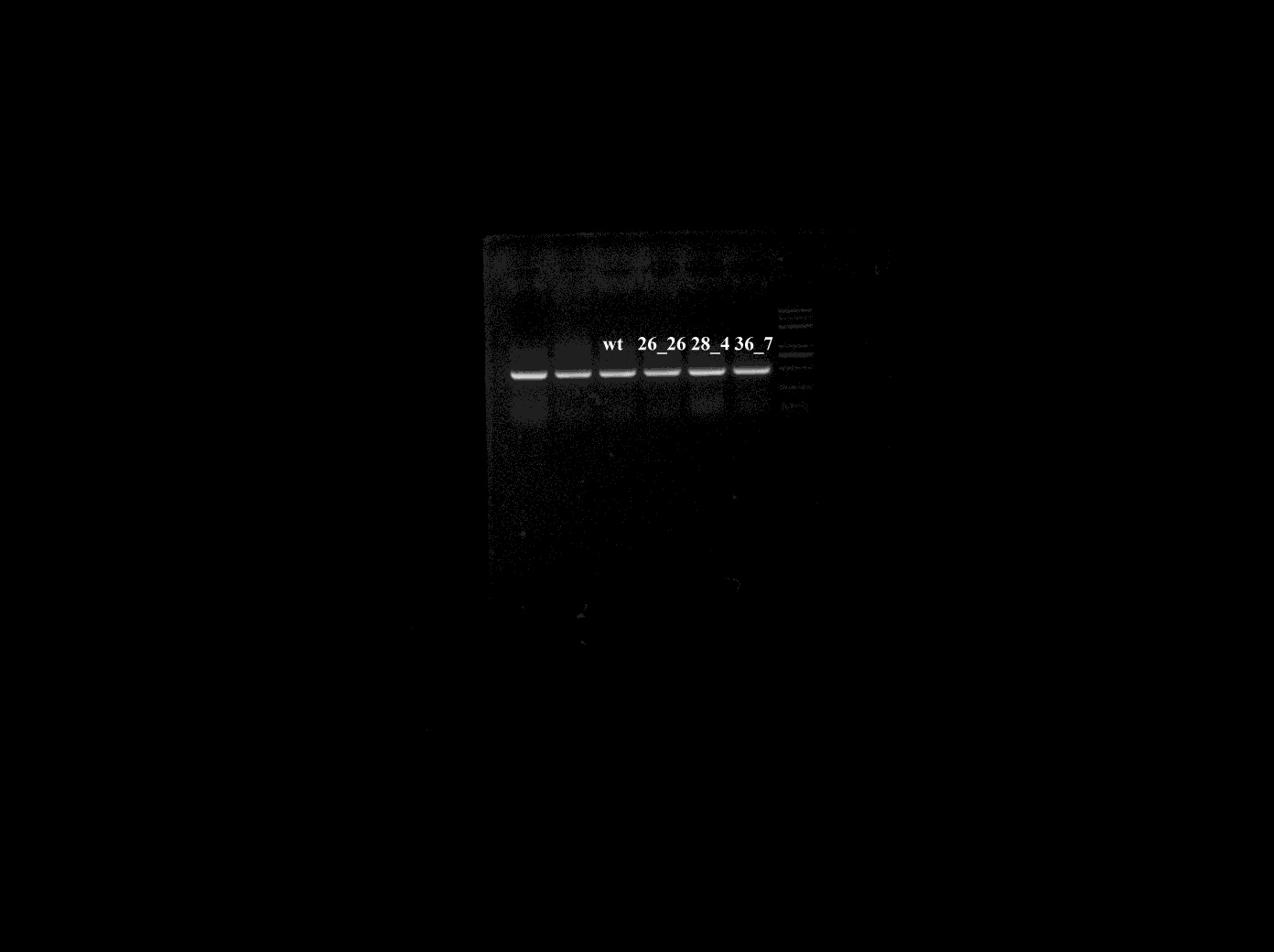

Supplement: Supplemental Information 3 — The β-actin blots of WT and three over-expressing lines are tagged with white words. [file peerj-08-8926-s007.jpg]

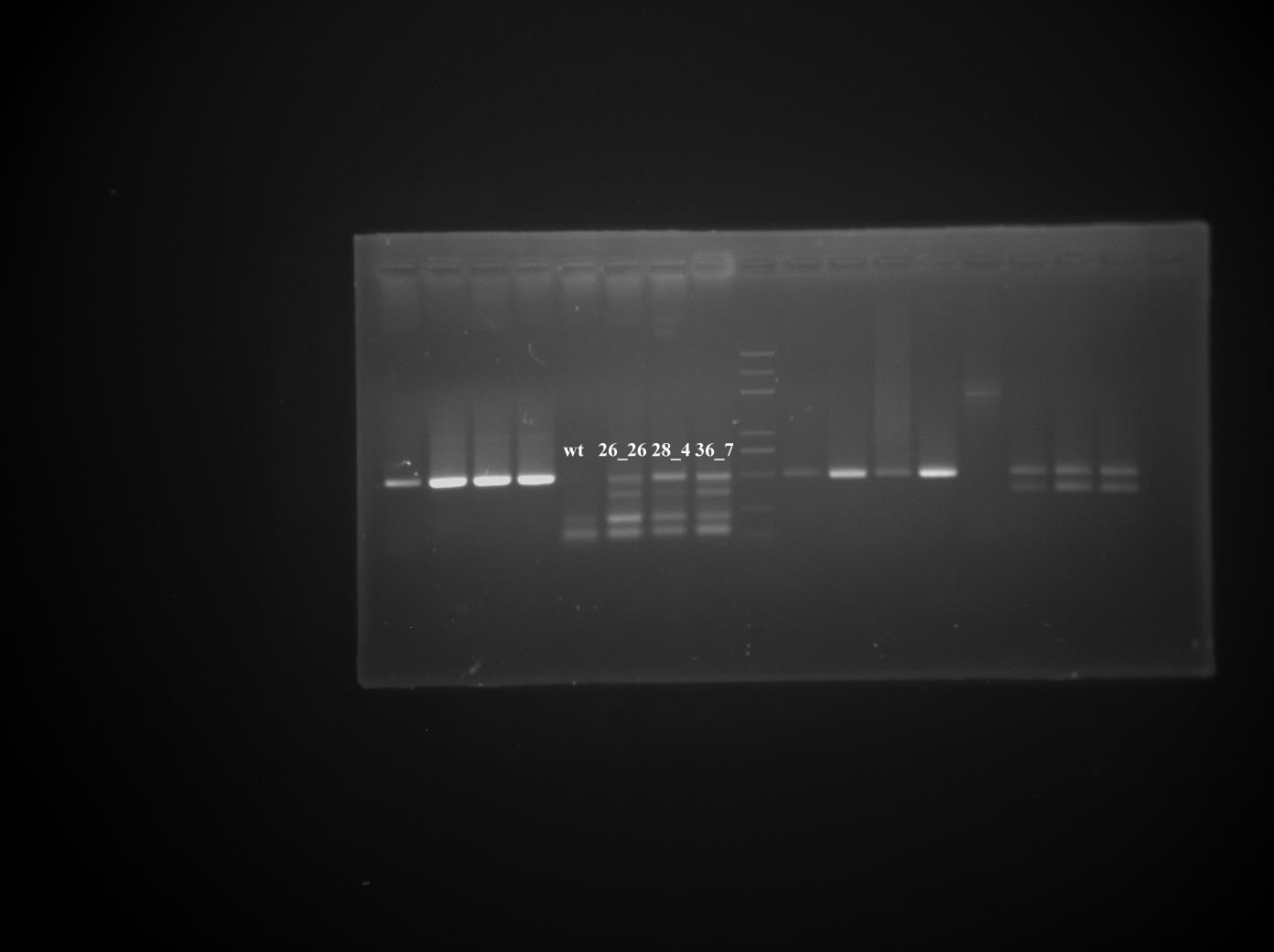

Supplement: Supplemental Information 4 — The ZmHsf01 blots of WT and three over-expressing lines are tagged with white words. [file peerj-08-8926-s008.jpg]
